# Supplementary figures and images for: Mutational processes of distinct POLE exonuclease domain mutants drive an enrichment of a specific TP53 mutation in colorectal cancer
Source: PLoS Genet. 2020 Feb 3;16(2):e1008572. doi: 10.1371/journal.pgen.1008572 (PMC7018097; doi:10.1371/journal.pgen.1008572)

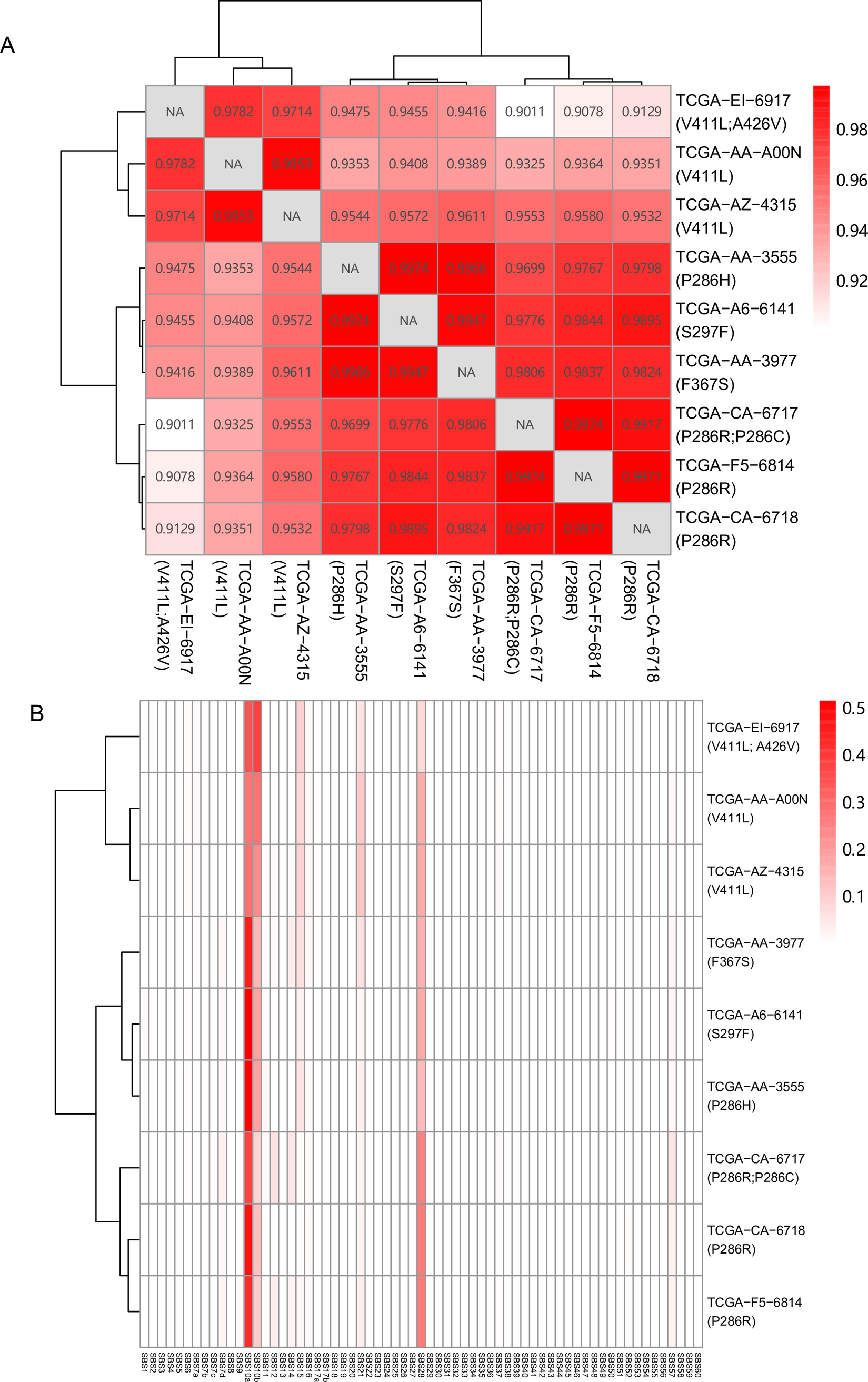

Supplement: S1 Fig — (A)Heatmap is based on cosine similarity as the value of cosine similarity is indicated in each cell. (B) Heatmap is based on COSMIC signature contribution, and each column represents one COSMIC signature. (TIF) [file pgen.1008572.s001.tif]

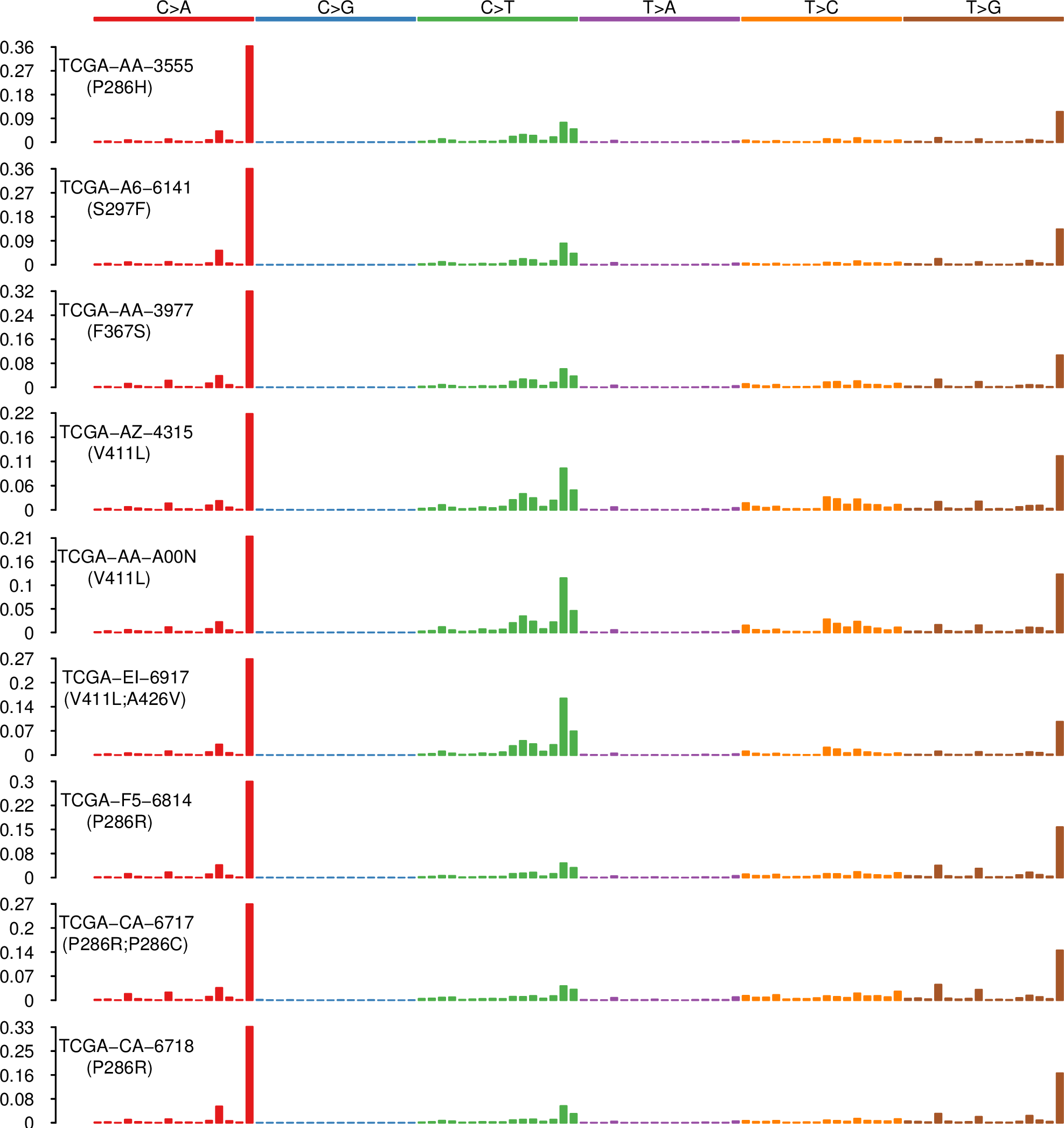

Supplement: S2 Fig — (TIF) [file pgen.1008572.s002.tif]

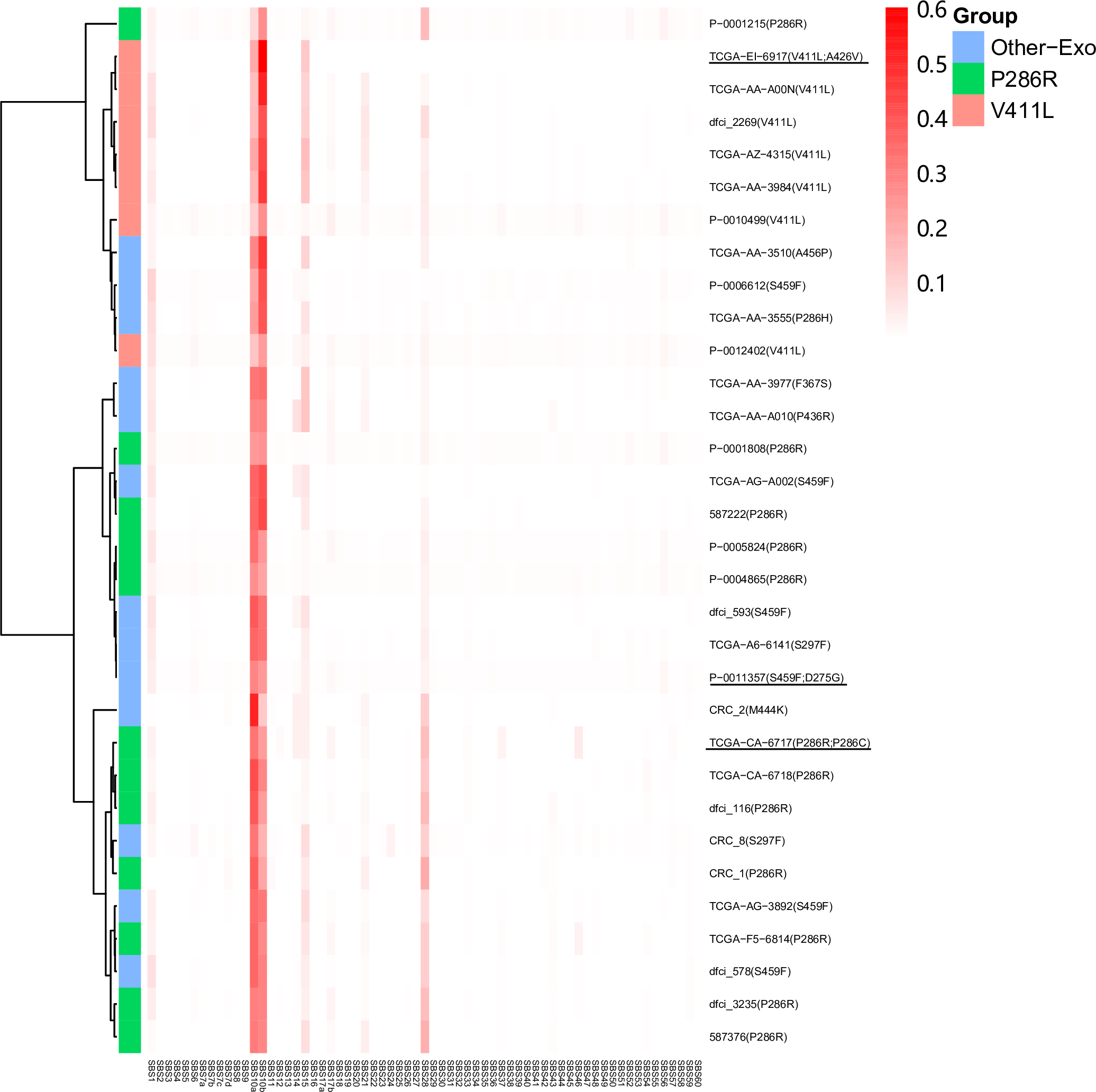

Supplement: S3 Fig — Samples with multiple POLE mutations are underlined. (TIF) [file pgen.1008572.s003.tif]

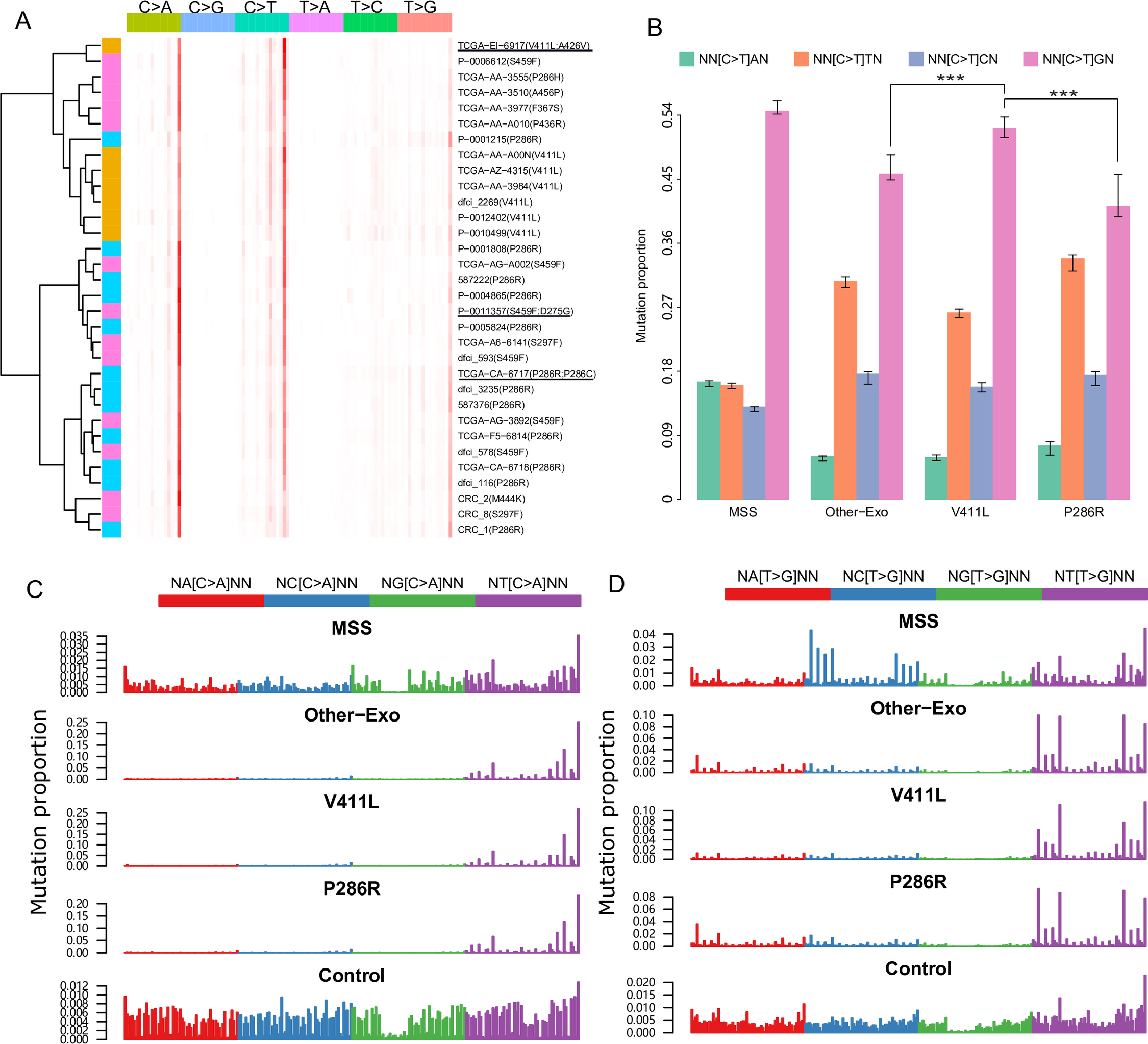

Supplement: S4 Fig — (A) Hierarchical clustered heatmap of the frequency of 96 types of mutational contexts for 32 POLE samples that have been whole genome, whole exome or targeted sequenced. Samples with multiple POLE mutations are underlined. (B) Proportion of C>T mutations in the CpA, CpC, CpG and CpT contexts. Profile of (C) C>A and (D) T>G (mutations in penta-nucleotide contexts, with genome-wide frequency of each penta-nucleotide indicated at bottom of each figure. (TIF) [file pgen.1008572.s004.tif]

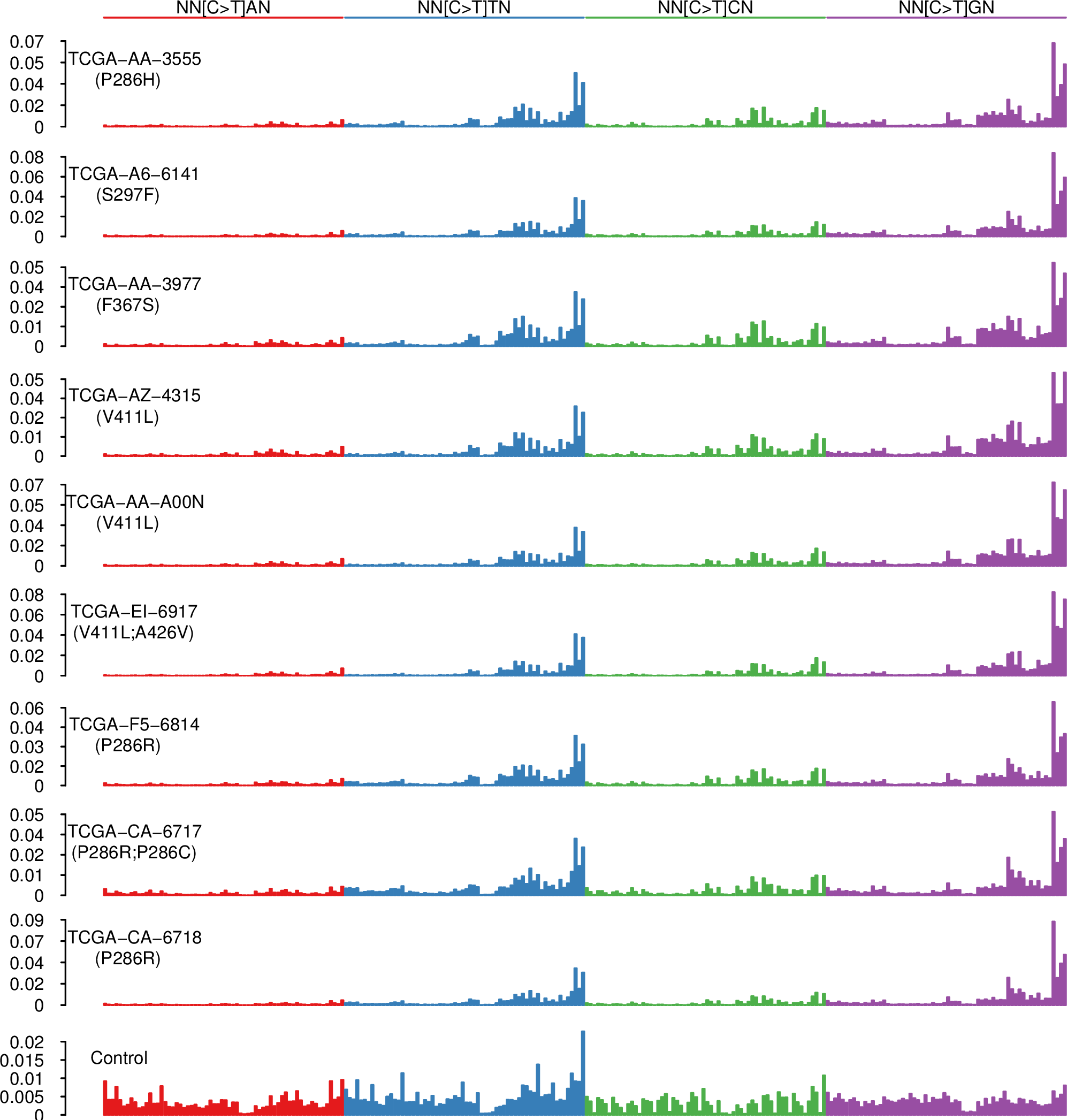

Supplement: S5 Fig — (TIF) [file pgen.1008572.s005.tif]

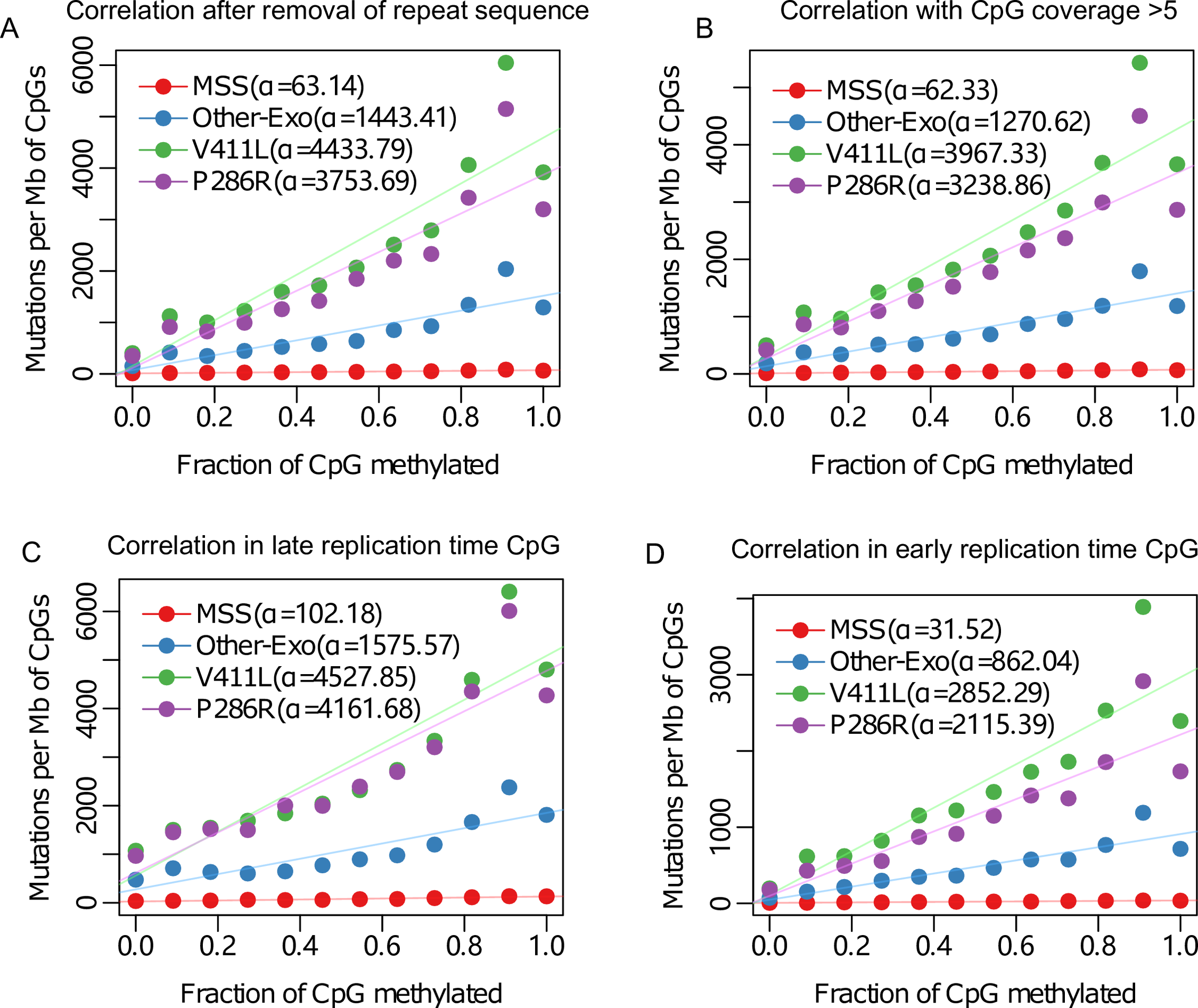

Supplement: S6 Fig — (A) Correlation of methylation and mutation burden after removal of repeat sequence in CpGs. (B) Correlation of methylation and mutation burden in the condition of the coverage of CpGs greater than five. (C) Correlation of methylation and mutation burden in late replication timing CpGs. (D) Correlation of methylation and mutation in early replication timing CpGs. (TIF) [file pgen.1008572.s006.tif]

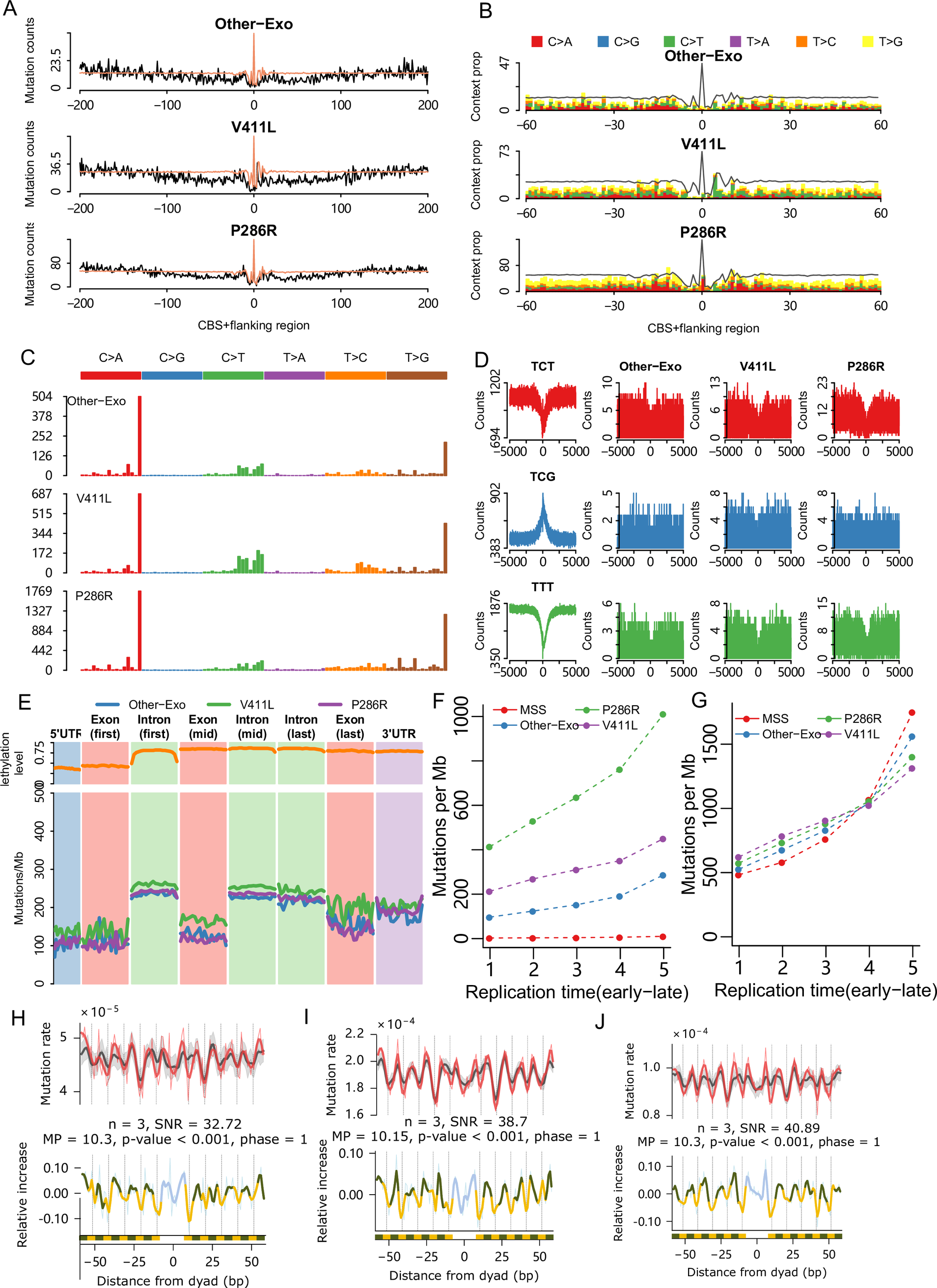

Supplement: S7 Fig — (A) Somatic substitutions at CBSs with a flanking sequence of 200 bp in different POLE mutants. The expected mutation was indicated in light red color. (B) Profile of mutation type was showed in CBSs with a flanking 200 bp sequence. (C) Mutational spectrum within ± 200bp sequence centered by CBS based on 96 mutational contexts. (D) Mutation profile around transcription start sites in different mutants. Three primary mutation types C>A, C>T and T>G in specific context were showed, and the abundance of each context was displayed in far left panel. (E) Profile of mutation burden across different parts of genes in different mutants. Mutation burden was normalized by the total number of mutations in each type of mutant. Association of mutational burden and replication timing. (F) DNA sequence with different replication timing was divided into 5 bins, and mutational burden was calculated in each bin ordered from early-to-late. (G) Mutational burden was normalized by total number of mutations in each type of mutant. Periodicity of tumor mutation rate within nucleosomes in different mutants: (H) Other-Exo, (I) V411L and (J) P286R. For each figure, the top panel shows observed and expected mutation rate, and the bottom panel shows relative increase of mutation rate. The bottom bar is schematic representation of alternating sequences of DNA with minor groove facing toward and away from histones. (TIF) [file pgen.1008572.s007.tif]

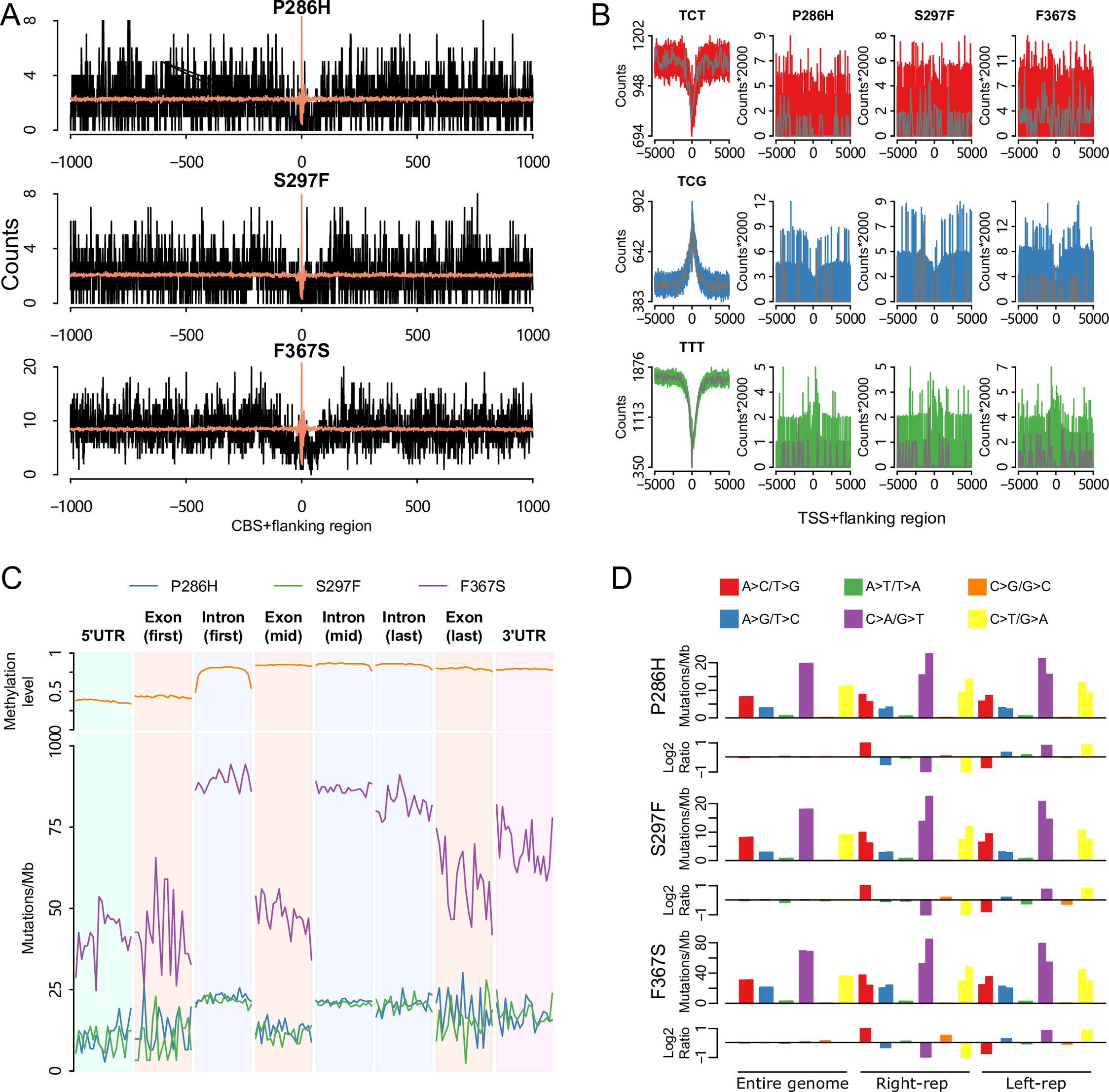

Supplement: S8 Fig — (A) Somatic substitutions at CBSs with a flanking sequence of 1 kilo bp in different POLE mutants. The expected mutation was indicated in light red color. (B) Mutation profile around transcription start sites in different mutants. Three primary mutation types C>A (red), C>T (blue) and T>G (green) in specific context were showed. Mutation counts were normalized by the number of corresponding context and the abundance of each context was displayed in the far left panel, together with mutation data in 100 bp bins (grey) is shown. (C) Profile of mutation burden across different part of genes in different mutants. Each part of gene was divided into 20 bins and mutation burden was calculated separately. Methylation level of each part was showed in the top panel. (D) Mutational strand asymmetry associated with replication in different mutants. Lower panel of each mutant shows the log2 ratio of each pair of bars. (TIF) [file pgen.1008572.s008.tif]

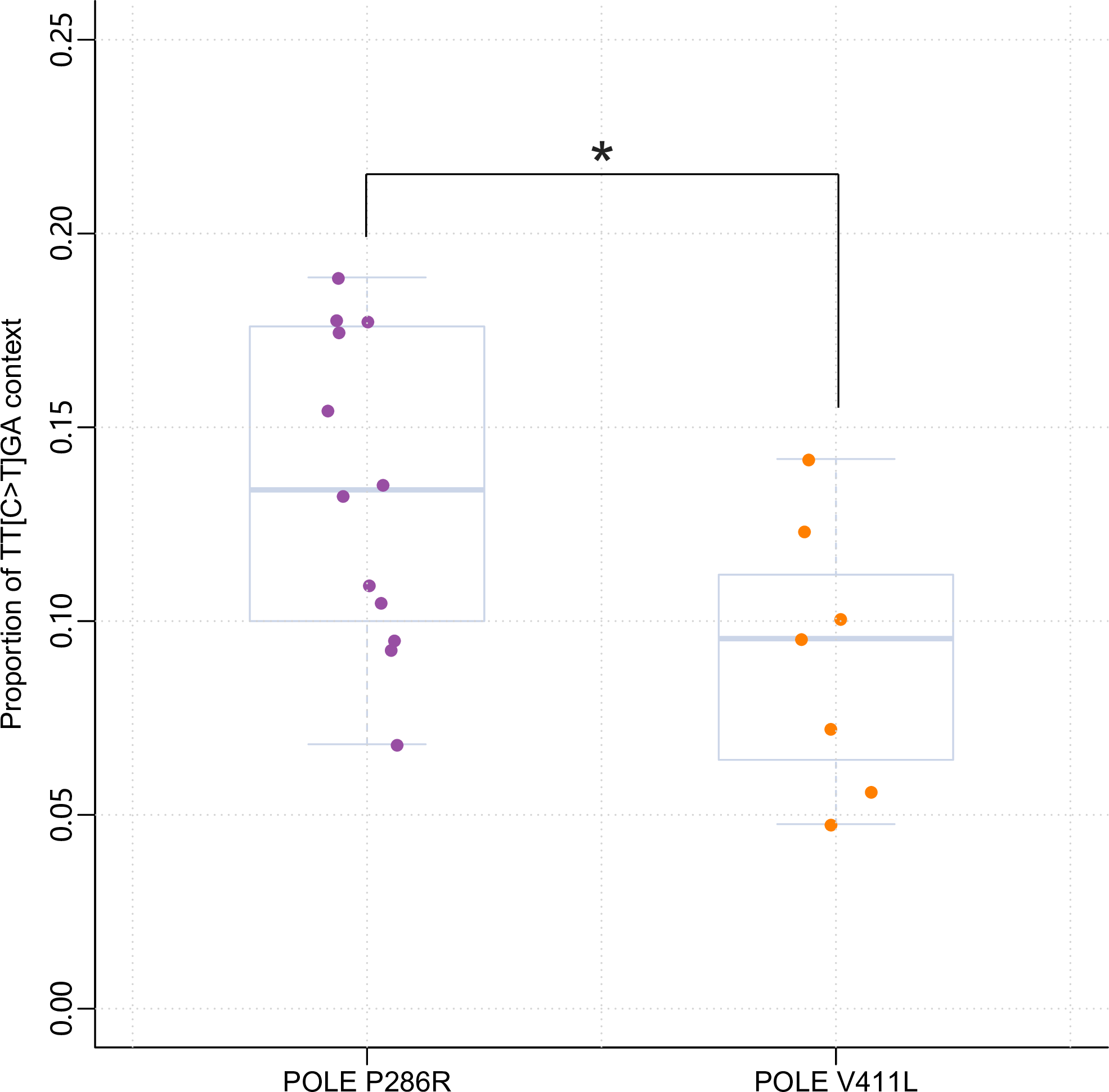

Supplement: S9 Fig — Only samples with total mutations to generate mutational contexts are included. * < 0.05, Student’s t-test. (TIF) [file pgen.1008572.s009.tif]
