# Supplementary material for: Mutational processes of distinct POLE exonuclease domain mutants drive an enrichment of a specific TP53 mutation in colorectal cancer
Source: PLoS Genet. 2020 Feb 3;16(2):e1008572. doi: 10.1371/journal.pgen.1008572 (PMC7018097; doi:10.1371/journal.pgen.1008572)
Supplement: Attachment — Submitted filename: Plos Gen Review-zp.docx [file pgen.1008572.s020.docx]

Tumors with mutations in POLE are among the most highly mutated tumors measured. The mutations occur in the exonuclease proofreading domain of the enzyme, but mouse and yeast model studies have raised interesting questions regarding the ultimate source of the mutagenesis. Studying the mutation burden and signatures from human patients has proven extremely valuable in understanding the etiologies of particular types of cancer.

In the current manuscript, Fang et al use bioinformatics and computational methods along with publicly available tumor sequencing data in an attempt to better understand the mutation signatures, and thus the origins of mutagenesis, in human POLE tumors. Based on the data presented, the authors conclude that: of the two highly recurrent mutant alleles, POLE V411L mutants have higher C>T mutagenesis than P286R; this imbalance is not dependent on CpG methylation status or several other genomic features; TP53-R213X nonsense mutations are specifically increased in P286R tumors due to a novel pentameric mutational signature error, TTCGA>TTTGA.

While several of the findings are suggestive, enthusiasm for the manuscript is dampened due to following issues.

• The TCG hotspot imbalance between P286R and V411L alleles has previously been observed and characterized by the Getz lab in a large cohort of POLE endometrial tumors (Haradhvala et al 2018). This study further distinguished TCG vs. TCT contribution on a per sample basis. This study was not cited by the authors.

• While the efforts of the authors to analyze a number of genomic features for their effects on mutagenesis is laudable, ultimately no role for any were observed.

• POLE mutations are quite abundant in endometrial cancer. This study would have benefitted immensely from analyzing that data alongside the colorectal data, helping confirm that their findings are POLE-specific not something tissue-specific.

• According to Fig. 1 and Supp. Table 1, TCGA CA-6717 (https://www.cbioportal.org/patient?studyId=coadread_tcga_pan_can_atlas_2018&caseId=TCGA-CA-6717) was indicated as one of the three WGS colorectal POLE P286R patient samples used. The POLE mutations in this sample, according to cBioportal, are I412L, L1235I and R1371X. According to their own Supp Table 1 it contains both P286R and P286S in addition to L1235I.

• The updated Cosmic mutation signature database now distinguishes 10a, 10b and 28 as distinct POLE signatures. As these each show different relative amounts of C>A-TCT, C>T-TCG and T>G-TTT mutations the current study would greatly benefit from reanalysis in this light.

• It is unclear why the authors used P286R n=12 and V411L n=7 to compare TTCGA context proportion in Supp. Fig 4 when there are n=16 and n=15 listed in Fig 5A.

• Fig. 1 describes the abundance of TCG mutations in V411L relative to P286R. Yet the specific TP53 mutation is a P286R-enriched TTCGA pentamer. This is hard to reconcile with the relatively modest increase in overall TTCGA mutagenesis in P286R shown in Supp Fig. 4 and is not addressed here.

• V411L tumors show increased MSI-L phenotype which may complicate the analyses here. It does not appear that the V411L samples were distinguished by MMR status.

Minor points

• The authors mention that the P286R amino acid change was predicted to affect the structure of the exonuclease binding pocket. This was actually also demonstrated structurally in the same issue (Parkash et al Nature Comm 2019). In addition to the above, there are a number of curious reference omissions. The 2012 TCGA study that first identified the POLE mutants in colorectal cancer is one. Another is the first reference. There is a vast body of literature on eukaryotic DNA polymerases and their fidelity that might have been used.

• The pentameric hotspots (e.g. Fig 1D and 3A) would greatly benefit from an inset with the individual sequence contexts clearly labeled.

• On balance the manuscript would benefit from more careful editing. Several examples through p. 6 include:

Line 50: “cause” should be “caused by”

Line 61: “understand” should be “understanding”

Line 89: “due to” is duplicated

Line 136: “Based” should be “Based on”
