# Supplementary material for: Mutational processes of distinct POLE exonuclease domain mutants drive an enrichment of a specific TP53 mutation in colorectal cancer
Source: PLoS Genet. 2020 Feb 3;16(2):e1008572. doi: 10.1371/journal.pgen.1008572 (PMC7018097; doi:10.1371/journal.pgen.1008572)
Supplement: Attachment — Submitted filename: Response_to_reviewers.docx [file pgen.1008572.s021.docx]

**Reviewer #1:**

Overall the study is well performed but some would argue predictable given some of the analyses in the original Shinbrot et al., paper, at least points 1 and 2 above, which are discussed in this paper (see Figures 3 and 6 of Shinbrot et al.,). That said I do feel that Fang et al., provides enhanced resolution and a refined analysis compared to Shinbrot et al. My only suggestion for the manuscript is that the authors provide a more critical comparison of their work to the paper of Shinbrot et al., to really bring out the novelty because at present this is not so obvious.

We thank the Reviewer for these suggestions. A more critical comparison of the analysis has now been provided as detailed below and also indicated in the revised manuscript.

1. In the Shinbrot et al. study, all POLE-exo mutants are divided into two groups based on the total mutational burden and C>A proportion. They demonstrated that mutants in Group A have defects in 3’-5’ exonuclease activity with a preference of C>A mutations. They however did not specifically compare mutational properties of different POLE mutants which is the focus of our study.

In our study, the first idea is to compare the different functional POLE mutants as several studies have reported that function or activity may vary due to the structure of catalytic site change (Parkash *et al.*, *Nat Commun* **2019;** Xing *et al.*, *Nat Commun* **2019**). We further classified the functional POLE (Group A in Shinbrot et al. study) mutants into different subgroups based on 96 trinucleotide mutational contexts by unsupervised clustering. We believe this is a more reasonable approach to group samples as it is better represented by the mutational properties across the whole genome (**Figure 1A**). The clusters are further supported by the panel data (**Supplementary Figure 4A**). We also fitted COSMIC signature database for each sample and obtained identical clusters based on the signature contribution (**Supplementary Figure 3**).

***Supplementary Figure 3***

*Hierarchical clustered heatmap of all POLE mutants based on COSMIC signature contribution. Samples with multiple POLE mutations are underlined.*

***Supplementary Figure 4A***

*Hierarchical clustered heatmap of the frequency of 96 types of mutational contexts for 32 POLE samples that have been whole genome, whole exome or targeted sequenced. Samples with multiple POLE mutations are underlined.*

2. Shinbrot et al. compared the potential nonsense sites and actual nonsense mutations in POLE mutants to demonstrate the mutation preference’s impact on nonsense enrichment in a specific gene (Shinbrot *et al.*, *Genome Res* **24**, 1740-1750, Figure 6), leading to the identification of recurrent nonsense mutations in key tumor suppressors such as *TP53, ATM*, and *PIK3R1*.

We again focused on the influence of mutation preference on hotspot in the different POLE exonuclease domain mutants. We first curated a list of recurrent mutations in 7,345 colorectal cancer samples and then tested each hotspot to see if they are enriched in any specific POLE mutant. To our knowledge, this is the largest cohort that are used to identify the association of hotspot and POLE mutants so far. From this cohort, we are able to compare the differences in driver mutation hotspots generated by different POLE mutants in a cancer type specific manner. This allowed us to show that the TP53 R213* is highly enriched in POLE P286R mutants.

3. We examined the relationship between DNA methylation and C>T mutation formation which was not addressed by the Shinbrot et al study. We show that the relative higher C>T mutations in V411L are independent on methylation, meaning that the increased relative frequency of these mutants in V411L compared with other mutants are due to the difference in rate in sequence contextual errors alone. We observed relative higher C>T mutations in V411L which are able to distinguish them from other mutants. Our study demonstrated that the methylation level could affect the C>T mutations in CpG dinucleotides in some extent, and the extra C>T mutations in V411L is independent (**Figure 2**).
4. The distribution of the large amount of mutations in different POLE mutants is explored genome-wide for the first time. As it is well established that POLE mutants are featured with high overall mutation incidence with elevated T[C>A]T and T[C>T]G, it is unclear how these mutations are distributed across the genome. We looked the different determinants that may affect mutation density across the whole genome, including CTCF binding sites, transcription start site, exonic and intronic regions, regions of different replication timing, regions across and within nucleosomes.

We summarize the comparison clarified above in our revised manuscript and have extensively revised our discussion to highlight these points, an excerpt of text is shown below (**Page 14**).

*Shinbrot et al. (2014) had previously characterized the functional POLE mutants with impaired exonuclease activity and describe a preference for C>A mutations in such mutants. Our study stratifies the functional POLE mutants in more detail based on the 96 trinucleotide mutational contexts and supported by expanded panel data (****Supplementary figure 1A****) and signature contribution (****Supplementary figure 2****). We show that higher frequency of C>T mutations in V411L mutants distinguishes them from other mutants. Methylated cytosine have been shown to readily mutate to thymine as a result of methylcytosine deamination (31). Although POLE V411L had comparably more mutations at the cytosine of CpG dinucleotides than other POLE exonuclease domain mutants (16.68% (V411L), 6.38% (P286R) and 10.19% (Other-Exo)), all mutants showed the same positive association with methylation level after adjusting for total CpG mutation count. This means that the higher level of C>T mutations in the POLE V411L compared with other POLE is not dependent on methylation, but rather sequence context is the major factor in determining the mutational spectrum of the different POLE exonuclease domain mutants. Our finding that different POLE mutants display consistent mutational distribution across genomic regions including CTCF binding sites, transcription start site, exonic and intronic regions, regions of different replication timing and regions across and within nucleosomes further confirms that the differential mutational process between POLE exonuclease mutants is principally at the localized sequence level.*

**Reviewer #2:**

1 Figure 1A, The mutational spectra was analyzed from 53 colorectal cancer whole genomes from TCGA and four types of signatures were identified in an hierarchical clustered heatmap. Please add Supplementary table 1 to the main manuscript, clearly showing the mutations in POLE for each of the nine samples (tumors) that was found in three groups. This is important since the study is focused on them from here on. After searching in the supplementary tables I found that 5 of the nine tumors carried more than one mutation in the exonuclease domain. How does that affect the downstream analysis? In case the mutations are located on the same allele of POLE then a second alteration anywhere in DNA polymerase epsilon can suppress or enhance the mutation rate and also affect the signature of the errors made by for instance P286R or V411L. Suppressors should be expected in case the mutation rate is very high during a period in the evolution of the cancer cells in the tumor and suppressors may affect the mutation signature.

We thank the Reviewer for this comment. We have now specified all the exonuclease domain mutations (268-471) of POLE for each group including samples with multiple mutations (**Figure 1A,** all other mutations have been listed in **Supplementary table 9**). We also annotated all other alterations in POLE in the WGS, WXS and targeted sequencing samples are now indicated in **Supplementary Figure 4A**.

To determine if secondary alterations in POLE might contribute to the mutational spectrum of the POLE mutant samples, we first calculated the cosine similarity for each mutant by using the proportion of 96 types of mutational context (**Supplementary Figure 1A**). We obtained identical clusters with **Figure 1A** based on cosine similarity value, indicating that samples within same cluster have similar mutational profile. Further, we fitted the well characterized COSMIC signatures for each mutant and performed the unsupervised cluster based on the signature contribution. We also obtained the identical clusters that consistent with mutational profile (**Supplementary Figure 1B**), suggesting the mutants within same cluster experienced similar mutational process.

***Supplementary Figure 1.*** *Hierarchical clustered heatmap of nine whole genome sequencing POLE mutants. (A)Heatmap is based on cosine similarity as the value of cosine similarity is indicated in each cell. (B) Heatmap is based on COSMIC signature contribution, and each column represents one COSMIC signature.*

Further, after excluding the mutations outside the exonuclease domain 268-471, only TCGA-EI-6917 (V411L; A426V) and TCGA-CA-6717 (P286R; P286C) have additional mutations beyond V411L and P286R. Tumors with A426V mutation have been reported with low mutational burden (< 10 Mut/Mb) and recognized as non-driver mutation (**Park et al, DNA Repair 2019**). For TCGA-CA-6717, P286R is caused by CCT > CGT and P286C is caused by CCT > TGT in which the CC> TG mutation is on the same allele (**Reviewer Response Figure 1**).

***Reviewer Response Figure 1.*** *Mapping of sequenced reads at chr12:133,253,184 in whole genome sequencing data and whole exome captured data.*

In summary, we conclude that none of the second alterations in these samples are likely to greatly impact or explain variation in the mutational spectrum beyond the established hotspot POLE mutations.

2 Please specify what the Other-exo variants are when first mentioned in the text. Grouping the Other-exo variants could mask any specific errors that could be made by individual POLE exo variants, if not show that this is not the case.

We thank the Reviewer for this suggestion. We have now specifically listed the POLE mutation in the Other-Exo variants in revised text and **Figure 1A.** It is true that different variants may have differential activity. We grouped them for following analysis based on these concerns: (1) They show relative more close mutational properties based the 96 types of mutational profile and cosine similarity (**Figure 1A; Supplementary Figure 1A**); (2) They experienced similar mutational process (**Supplementary Figure 1B)**.

We now carried out the analysis of the samples in Other-Exo group individually as indicated in **Supplementary Figure 8.**

The results we obtained from individual sample don’t affect the interpretation of the main conclusions as all mutants show very consistent mutational patterns in terms of CTCF binding sites, transcription start site, exonic and intronic regions and mutational asymmetry. We have now added this as **Supplementary Figure 8** in the manuscript and the follow text on **page 11-12.**

*“In order to investigate if the individual samples in the Other-Exo group could mask any specific errors, we carried out the analysis of the samples in Other-Exo group individually (****Supplementary Figure 8).*** *All mutants show very consistent mutational patterns in terms of CTCF binding sites, transcription start site, exonic and intronic regions and mutational asymmetry.”*

***Supplementary Figure 8*** *Genome-wide mutational patterns of distinct POLE mutants in Other-Exo subgroup. (****A****) Somatic substitutions at CBSs with a flanking sequence of 1 kilo bp in different POLE mutants. The expected mutation was indicated in light red colour. (****B****) Mutation profile around transcription start sites in different mutants. Three primary mutation types C>A (red), C>T (blue) and T>G (green) in specific context were showed. Mutation counts were normalized by the number of corresponding context and the abundance of each context was displayed in the far left panel, together with mutation data in 100 bp bins (grey) is shown. (****C****) Profile of mutation burden across different part of genes in different mutants. Each part of gene was divided into 20 bins and mutation burden was calculated separately. Methylation level of each part was showed in the top panel. (****D****) Mutational strand asymmetry associated with replication in different mutants. Lower panel of each mutant shows the log2 ratio of each pair of bars.*

3 I would like to see that tumors with more than one altered amino acid in the exonuclease domain is removed from the sample set. An alternative could be to show that the mutation spectra in such a sample is identical to when only P286R or V411L is present. It may also be acceptable if the authors exclude the possibility that the POLE variants are not located on the same allele.

We have indicated the POLE mutants with multiple mutations within exonuclease domain (**Supplementary Figure 4A**). For all seven mutants with V411L variant, only one of them has additional variant (TCGA-EI-6917: A426V), and this variant has been reported as non-driver mutation (**Park et al, DNA Repair 2019**). For all 13 P286R mutants, only one has another mutation (TCGA-CA-6717: P286C) which is caused by CCT > TGT change, and it is located on the same allele with P286R (**Reviewer Response Figure 1**). These two variants also have been addressed in the response to the first comment.

4 Are all samples in Figure 1A mismatch repair proficient? If not how are mismatch repair deficient samples divided among the four groups in in Figure 1A

The microsatellite status of all samples in figure 1A are indicated in **Supplementary table 1**. Only one sample TCGA-AA-A00N show MSI-L and all the others are MSS.

5 Is MSS and V411L significantly different from each other in Figure 1C? If not, why are they clustered differently in Figure 1 A? I would also like to know whether P286R and Other-Exo are significantly different in figure 1C.

Generally, MSS (no POLE mutation) samples and POLE mutants have totally different profile as their mutational process is very different. Although the frequency of C>T mutations for MSS and V411L POLE mutants are not significantly different (**Reviewer Response Table 1)**, the difference, at the trinucleotide level, is profound and highly significant (**Reviewer Response Figure 2, Reviewer Response Table 2**). Since **Figure 1A** is based on trinucleotide frequencies, that is why this is reflected in the differential clustering of MSS and V411L. Furthermore, as we can see from **Figure 1B,** MSS sample presents distinct proportion of 96 types of mutational context when compared with other POLE mutants.

For P286R versus Other-Exo, the difference is significant both at the single nucleotide level (**Reviewer Response Table 1**) as well as at the trinucleotide level (**Supplementary Figure 1A).**

***Reviewer Response Table 1****. Chi-squared test of C>T mutation between MSS and V411L, P286R and Other-Exo.*

| P= 0.2726 (Chi-squared test) | **C>T** | **Non C>T** |
| --- | --- | --- |
| **V411L** | 917233 | 1801719 |
| **MSS** | 229312 | 451858 |

| P < 2.2e-16 (Chi-squared test) | **C>T** | **Non C>T** |
| --- | --- | --- |
| **P286R** | 914252 | 4809557 |
| **Other-Exo** | 339518 | 1116958 |

***.***

***Reviewer Response Table 2.*** *T- test of different C>T mutational context between MSS and V411L.*

***Reviewer Response Figure 2.*** *Proportion of C>T mutation in MSS and V411L samples. The tri-nucleotide contexts where the C>T mutation located are indicated in the bottom. Error bars represent +/- 2 SE.*

6 Lane 245, I do not understand how the mutation rate can be calculated since the number of cell divisions are unknown. Could you please explain how the mutation rate is calculated.

We apologise for this confusion, we should have described this as “mutation density” rather than “mutation rate”. The mutation density for each region is calculated by: (total mutation number)/(size of replicated region). Regions of different replication timing was obtained for the HepG2 cell line from the ENCODE data portal. This has now been corrected throughout the manuscript.

7 Lane 355-362, Could you please explain why the TP53 hotspot is significantly enriched in POLE P286R mutants, but not in POLE V411L mutants although the POLE V411L mutants have a significantly higher C>T transition frequency when compared to POLE P286R ?

We thank the Reviewer for this comment and agree that it should be discussed more thoroughly. Firstly, we have shown in **Figure 5B**, TP53 R213* specifically occurs in a TT[C>T]GA pentanucleotide context. Although POLE V411L has overall higher C>T transition frequency compared with POLE P286R, this particular TT[C>T]GA context of R213* is favoured in POLE P286R when considering C>T in CpG sites (**Figure 5C, D** and **Supplementary figure 9**). Moreover, if we consider all TTCGA sites in the genome, such sites are 15% more likely to have a C>T mutation in POLE P286R compared with V411L (**Supplementary table 5**). Furthermore, as TP53 mutations are under strong positive selection in MSS colorectal cancer, it is likely to reinforce the enrichment of the TP53 R213* mutations in POLE P286R. We have modified Figure 5 to more clearly illustrate this result and also revised the results and discussion section as outlined below:

**Figure 5.** Mutation hotspots in POLE mutants. (A) Contingency table of different POLE-mutant and POLE wild type colorectal cancer samples with or without the TP53 R213* mutation. Samples with Sig10 were confirmed by either POLE driver mutation or mutational spectrum clustering. (B) Truncating mutation TP53 R213* was caused by C>T substitution in TT[C>T]GA context. (C) Frequency of 21-bp sequence context centered by mutated cytosine in different POLE mutants, and the penta-nucleotide contexts were indicated in black box. Proportion of TT[C>T]GA mutations in the NN[C>T]GN pentanucleotide context in all POLE P286R and V411L mutants (D) and (E) POLE P286R mutants with and without the TP53 R213* mutation. * < 0.05. ** < 0.01, Student’s t-test.

Results

*“This nonsense mutation is a C>T transition in the context of TT[C>T]GA (****Figure 5B****), which is a relatively enriched context in P286R mutants, with adenine being more prevalent in the 5^th^ position, compared with the other POLE mutants (****Figure 5C,*** *p <0.05, Student’s t-test,* ***Supplementary figure 9)****. Since the mutation also occurs at a highly methylated CpG site (20), we specifically compared the frequency of TT[C>T]GA in P286R versus V411L relative to the number of C>T mutations at CpG sites (i.e. NN[C>T]GN). It is evident that this context is significantly more enriched in POLE P286R mutants (Figure 5D, p < 0.01, Student’s t-test). To quantify the effect this difference might have on the mutagenesis of TTCGA sites across the genome, we counted the number of sites in POLE P286R, V411L and other exonuclease domain mutants respectively and normalised the count by the total number of NN[C>T]GN mutations in each group (****Supplementary Table 5****). We find that such sites are 15% more likely to be mutated (5.68% versus 4.95% of TTCGA mutated in P286R and V411L respectively). As the relative frequency of the TT[C>T]GA pentanucleotide context differs between individual samples, we also compared its relative frequency in POLE mutants with and without the TP53 R213* mutation. This mutational context was found to not only be significantly higher in POLE P286R mutants with the TP53 R213* mutation compared with those without this mutation (P <0.05, Student’s t-test,* ***Figure 5E****). This suggests that for C>T mutations, POLE mutant colorectal cancers (and in particular the P286R mutant) that generate relatively more mutations in the TT[C>T]GA context, have a higher chance of acquiring the TP53 R213* mutation.”*

*--*

Discussion

*“The TP53 R213* truncating mutation is caused by a C>T transition in a TTCGA penta-nucleotide context, and we found that POLE mutants with this mutation do generally have higher relative frequency of this mutational context compared to POLE exonuclease domain mutants without this mutation. This implies a possible direct causal relationship between POLE-associated mutagenesis and acquisition of this driver mutation. However, although the difference in the relative mutation frequency between POLE R286R and the other exonuclease domain mutations is significant, this difference in level is modest (~15%) and thus the selection for TP53 mutations may also play a role in the final observed frequency of such mutations.”*

Minor points

8 Lanes 53-55, “Previous cancer genomics studies have identified a number of mutation hotspots in POLE, however how these different POLE mutants behave in shaping the mutational landscape of cancers has not been studied.” I think this is an over-statement. Please read the review and references therein by Park and Pursell, DNA repair, title: POLE proofreading defects: Contributions to mutagenesis and cancer.

We thank the Reviewer for this comment. We intended to say that the distribution of mutations across the whole genome has not been studied. To avoid misunderstanding, we revised it as (**Page 3**):

*“Previous cancer genomics studies have identified a number of mutation hotspots in POLE, however how these different POLE mutants behave in affecting mutation distribution across the genome has not been studied”.*

9 Lane 82, “Residue P286 is located in the DNA binding pocket which is adjacent to the exonuclease active site. A change of this amino acid has been predicted to affect the structure of the DNA binding pocket and cause polymerase hyperactivity (6).” It was earlier a prediction, but it was recently shown to be the case in a high resolution structure by Parkash et al in Nature Comm 2019. In fact, Xing et al in the companion paper show that the P to R subsititution cause the enzyme to become hyperactive and has an increased capacity to extend mismatches.

We have revised the related section as shown in the excerpt of text below (**Page 4**).

*“The POLE mutational process shapes the cancer genome into a unique mutational signature with high proportions of C>A mutations at TCT contexts, C>T mutations at TCG contexts and T>G mutations at TTT contexts, which is known as COSMIC signature 10 (4). Several driver mutations have been identified in the POLE exonuclease domain (codons 268–471) (5), the most frequent being P286R and V411L (2). The crystal structure of the yeast orthologue have showed that P301R (P286R in Human) could change the exonuclease domain, with R301 pointing towards the exonuclease site, leading to polymerase hyperactivity and increased capacity to extend mismatches by interfering with DNA binding to the exonuclease site(6,7). By contrast, residue V411 lies a distance away from the binding site and does not interact with the DNA sequence directly (8). Data showing the mutation spectrum of individual POLE mutants supports differences in the way mutants generate somatic mutations (9) but these differences have not yet been quantified.”*

10 Lane 89, typo, “due to” is written twice in a row, “due to due to”

Modified.

11 Lane 308, “P286 lies in the DNA binding pocket, which might interact with single strand DNA by directly perturbing the binding pocket.” Again, please see Parkash et al Nature Comm, 2019 that specifically show and discuss this point.

We thank the Reviewer for this comment. We have discussed this issue more in detail in the text. The revised excerpt is shown as below (**Page 17**).

*“V411L and P286R are the two most frequent POLE mutants and they are located far away from each other in the exonuclease domain, thus conferring different functions (29). Structural and molecular dynamics simulation studies in S. cerevisiae have revealed that P301R (P286R in Human) substitution prevents proper positioning of ssDNA in the exonuclease active site of Pol ε, resulting in promoting the extension of mismatched primer termini (6,7). However, V411 is far away from exonuclease active site may function by affecting the positions of other residues adjacent to the active site (8).”*

**Reviewer #3:**

1 The TCG hotspot imbalance between P286R and V411L alleles has previously been observed and characterized by the Getz lab in a large cohort of POLE endometrial tumors (Haradhvala et al 2018). This study further distinguished TCG vs. TCT contribution on a per sample basis. This study was not cited by the authors.

We thank the Reviewer for the comment. In Haradhvala et al. study, P286R and V411L mutants are distinguished by signature contribution (**Haradhvala et al. Nature comm 2018,** **Fig 2C**). V411L mutants show high fraction of E3 which has elevated T[C>T]G mutations. We now have cited this study in introduction section as follows (**Page 4**):

*“By contrast, residue V411 lies a distance away from the binding site and does not interact with the DNA sequence directly (9). In endometrial cancer, it has been shown that V411L and P286R display different signature fraction with V411L is characterized by relative higher fraction of C>T mutations in endometrial cancer (10). Data showing the mutation spectrum of individual POLE mutants supports differences in the way mutants generate somatic mutations (11) but these differences have not yet been quantified.*

2 While the efforts of the authors to analyze a number of genomic features for their effects on mutagenesis is laudable, ultimately no role for any were observed.

Although we did not find differences in the way the different POLE exonuclease domain mutants mutate the genomic features we analyzed, we want to emphasize that this finding is still important because it shows that the main difference between the different mutants is exclusively at the local sequence level. Significantly, although it has been previously shown that methylation plays a major role in drive C>T mutations (**Poulos et al 2017, Tomkova et al. 2018**), we demonstrate that this is not a factor in the relatively higher frequency of C>T mutations in V411L compared with P286R (**Figure 2B**). This is not trivial as this relationship has never previously been demonstrated. We have now highlighted this more clearly in the Discussion section:

*“We show that higher frequency of C>T mutations in V411L mutants distinguishes them from other mutants. Methylated cytosine have been shown to readily mutate to thymine as a result of methylcytosine deamination (31). Although POLE V411L had comparably more mutations at the cytosine of CpG dinucleotides than other POLE exonuclease domain mutants (16.68% (V411L), 6.38% (P286R) and 10.19% (Other-Exo)), all mutants showed the same positive association with methylation level after adjusting for total CpG mutation count. This means that the higher level of C>T mutations in the POLE V411L compared with other POLE is not dependent on methylation, but rather sequence context is the major factor in determining the mutational spectrum of the different POLE exonuclease domain mutants. Our finding that different POLE mutants display consistent mutational distribution across genomic regions including CTCF binding sites, transcription start site, exonic and intronic regions, regions of different replication timing and regions across and within nucleosomes further confirms that the differential mutational process between POLE exonuclease mutants is principally at the localized sequence level.”*

3 POLE mutations are quite abundant in endometrial cancer. This study would have benefitted immensely from analyzing that data alongside the colorectal data, helping confirm that their findings are POLE-specific not something tissue-specific.

We thank the Reviewer for this suggestion. We have now looked at 2045 endometrial cancer samples across three different cohorts (**Supplementary table 5**). We found that the TP53 R213* is enriched in endometrial POLE mutants as well (**supplementary table, p-value<0 .001,** **chi-square test, Supplementary table 6**). However, in contrast to colorectal cancer this is not enriched in any specific mutant. We suspect that this may relate to the higher level of positive selection for TP53 mutations in colorectal cancer compared with endometrial cancers (see response to point 7 below). We have added this result in revised manuscript (**Page 13**). As follows:

*To determine if this effect is cancer specific, we also explored if the enrichment of this hotspot is present in 2045 endometrial cancer samples (****Supplementary table 5****). TP53 R213* is not more enriched in POLE P286R mutants than other POLE mutants but it is significantly enriched in endometrial POLE mutants (P<0.001, Chi-square test,* ***Supplementary table 6****) as 15.28 % (11/72) POLE mutants harbor this hotpot while it is identified in 0.006% (11/1973) non-POLE mutants. This suggests that this enrichment is specific to colorectal cancer and may reflect the higher apparent positive selection for TP53 mutations in colorectal cancer compared with endometrial cancers in general with 57% (4,677/7,345) TP53 mutants in colorectal cancer versus 48% (974/2,045) TP53 mutants in endometrial cancer (P < 0.001, Fisher's exact test).*

4 According to Fig. 1 and Supp. Table 1, TCGA-CA-6717 (https://www.cbioportal.org/patient?studyId=coadread_tcga_pan_can_atlas_2018&caseId=TCGA-CA-6717) was indicated as one of the three WGS colorectal POLE P286R patient samples used. The POLE mutations in this sample, according to cBioportal, are I412L, L1235I and R1371X. According to their own Supp Table 1 it contains both P286R and P286S in addition to L1235I.

Thank you for the comment. The data used for TCGA-CA-6717 in this study is whole genome sequencing data. We checked the bam file of whole genome sequencing (WGS) data and whole exome sequencing (WES) data to confirm the mutation in POLE manually as indicated in (**Reviewer Response Figure 1; Reviewer Response Figure 4**). I412L is a mutation in POLE2 not POLE (See **Reviewer Response Figure 3**).

***Reviewer Response Figure 3.*** *POLE mutations in sample TCGA-CA-6717 from cBioportal.*

By examining the BAM files, we observed P286R, P286C, L1235I and R1371X in both WGS and WES data. According to the mapping result, P286R is caused by CCT > CGT and the other one is actually P286C caused by CCT > TGT (see **Reviewer Response Figure 1)**.

***Reviewer Response Figure 4*** *Mapping of sequenced reads at chr12:133,226,355 and chr12:133,225,553 in whole genome sequencing data and whole exome captured data.*

5 The updated Cosmic mutation signature database now distinguishes 10a, 10b and 28 as distinct POLE signatures. As these each show different relative amounts of C>A-TCT, C>T-TCG and T>G-TTT mutations the current study would greatly benefit from reanalysis in this light.

We thank the Reviewer for this good point. We have added this analysis in the manuscript as indicated in **(Supplementary Figure 1B; Supplementary Figure 3)**.

6 It is unclear why the authors used P286R n=12 and V411L n=7 to compare TTCGA context proportion in Supp. Fig 4 when there are n=16 and n=15 listed in Fig 5A.

We apologize for this confusion. In Fig 5A, we included all the samples as long as TP53 and POLE mutations are available. But for the **supp. Fig 4 (revised Supplementary Figure 9)**, we need all mutations of each sample to generate mutational context. But for samples in cohorts of “GENIE” and “Helsinki_University”, only mutations in hotspot genes are available, which is indicated in **Supplementary table 7.** We have added the following sentence to the legend of **Supplementary Figure 9** to clarify this:

*“Proportion of C>T mutations in the TTCGA pentanucleotide context in POLE P286R mutants and POLE V411L mutants. Only samples with total mutations to generate mutational contexts are included. * < 0.05, Student’s t-test.”*

7 Fig. 1 describes the abundance of TCG mutations in V411L relative to P286R. Yet the specific TP53 mutation is a P286R-enriched TTCGA pentamer. This is hard to reconcile with the relatively modest increase in overall TTCGA mutagenesis in P286R shown in Supp Fig. 4 and is not addressed here.

We thank the Reviewer for this comment. We agree that the difference between the pentamer enrichment in absolute terms is not very large. However, if we consider that the TP53 R213* occurs at a highly methylated CpG site, then relative to [C>T]pG mutations, the enrichment of P286R is significant compared with V411L (Figure 5D). We have now performed additional analyses to show that across all TTCGA sites in the genome, such sites are 15% more likely to have a C>T mutation in POLE P286R compared with V411L. We have now also discussed the role of selection in the enrichment of the TP53 R213* hotspot. TP53 mutations are under strong positive selection in colorectal cancer. This selection may reinforce the enrichment of the TP53 R213* mutations in POLE P286R. As TP53 mutations are more prevalent in colorectal cancer compared with endometrial cancer, this may also explain why the enrichment of TP53 R213* in POLE P286R mutants is specific in colorectal cancer. We modified Figure 5 and add the following to results (**Page 12-13**) and discussion section (**Page 16**):

**Figure 5.** Mutation hotspots in POLE mutants. (A) Contingency table of different POLE-mutant and POLE wild type colorectal cancer samples with or without the TP53 R213* mutation. Samples with Sig10 were confirmed by either POLE driver mutation or mutational spectrum clustering. (B) Truncating mutation TP53 R213* was caused by C>T substitution in TT[C>T]GA context. (C) Frequency of 21-bp sequence context centered by mutated cytosine in different POLE mutants, and the penta-nucleotide contexts were indicated in black box. Proportion of TT[C>T]GA mutations in the NN[C>T]GN pentanucleotide context in all POLE P286R and V411L mutants (D) and (E) POLE P286R mutants with and without the TP53 R213* mutation. * < 0.05. ** < 0.01, Student’s t-test.

Results

*“This nonsense mutation is a C>T transition in the context of TT[C>T]GA (****Figure 5B****), which is a relatively enriched context in P286R mutants, with adenine being more prevalent in the 5^th^ position, compared with the other POLE mutants (****Figure 5C,*** *p <0.05, Student’s t-test,* ***Supplementary figure 9)****. Since the mutation also occurs at a highly methylated CpG site (20), we specifically compared the frequency of TT[C>T]GA in P286R versus V411L relative to the number of C>T mutations at CpG sites (i.e. NN[C>T]GN). It is evident that this context is significantly more enriched in POLE P286R mutants (Figure 5D, p < 0.01, Student’s t-test). To quantify the effect this difference might have on the mutagenesis of TTCGA sites across the genome, we counted the number of sites in POLE P286R, V411L and other exonuclease domain mutants respectively and normalised the count by the total number of NN[C>T]GN mutations in each group (****Supplementary Table 5****). We find that such sites are 15% more likely to be mutated (5.68% versus 4.95% of TTCGA mutated in P286R and V411L respectively). As the relative frequency of the TT[C>T]GA pentanucleotide context differs between individual samples, we also compared its relative frequency in POLE mutants with and without the TP53 R213* mutation. This mutational context was found to not only be significantly higher in POLE P286R mutants with the TP53 R213* mutation compared with those without this mutation (P <0.05, Student’s t-test,* ***Figure 5E****). This suggests that for C>T mutations, POLE mutant colorectal cancers (and in particular the P286R mutant) that generate relatively more mutations in the TT[C>T]GA context, have a higher chance of acquiring the TP53 R213* mutation.*

*To determine if this effect is cancer specific, we also explored if the enrichment of this hotspot is present in 2045 endometrial cancer samples (****Supplementary table 5****). TP53 R213* is not more enriched in POLE P286R mutants than other POLE mutants but it is significantly enriched in endometrial POLE mutants (P<0.001, Chi-square test,* ***Supplementary table 6****) as 15.28 % (11/72) POLE mutants harbor this hotpot while it is identified in 0.006% (11/1973) non-POLE mutants. This suggests that this enrichment is specific to colorectal cancer and may reflect the higher apparent positive selection for TP53 mutations in colorectal cancer compared with endometrial cancers in general with 57% (4,677/7,345) TP53 mutants in colorectal cancer versus 48% (974/2,045) TP53 mutants in endometrial cancer (****P < 0.001****, Fisher's exact test).”*

*--*

Discussion

*“However, although the difference in the relative mutation frequency between POLE R286R and the other exonuclease domain mutations is significant, this difference in level is modest (~15%) and thus the selection for TP53 mutations may also play a role in the final observed frequency of such mutations. Furthermore, the enrichment of TP53 R213* was not evident in POLE P286R mutant endometrial cancer. As TP53 mutations are more prevalent in colorectal cancers compared with endometrial cancers, this may also suggest that stronger selective pressure for such mutations is required for this observation in POLE P286R colorectal cancers.”*

8 V411L tumors show increased MSI-L phenotype which may complicate the analyses here. It does not appear that the V411L samples were distinguished by MMR status.

In the revised manuscript, the microsatellite status of all POLE mutants has been summarized in **Supplementary table 1**. For cohorts with microsatellite available in our study, only TCGA cohort has the microsatellite status classified as MSS, MSI-L and MSI, for the other cohorts, they have only been classified as MSS and MSI. We could not show that the V411L mutants show increased MSI-L phenotype from the current data available (**Reviewer Response Table 3**) but rather they likely clustered together due to the distinct mutational profile (**Supplementary Figure 4A**). We checked TCGA colorectal V411L samples used in this study, only one (TCGA-AA-A00N) was labeled as MSI-L. More data are needed to demonstrate the association of V411L and MSI-L in colorectal cancer.

**Haradhvala *et al.*,** mentioned that “POLE-exo* tumors with a V411L mutation were often classified as MSI-L” in endometrial cancer. We also checked the microsatellite status in endometrial POLE mutants as indicated in **Reviewer Response Table 4** It still can’t make a conclusion that V411L mutants show significant higher MSI-L phenotype (Pearson's Chi-squared test, P= 0.08632).

***Reviewer Response Table 3****. Contingency table comparing microsatellite status in colorectal cancer.*

|  | **MSI-H** | **MSI-L** | **MSS** | **NA** |
| --- | --- | --- | --- | --- |
| **Other-Exo** | 0 | 1 | 11 | 4 |
| **P286R** | 1 | 0 | 11 | 4 |
| **V411L** | 1 | 1 | 5 | 8 |

***Reviewer Response Table 4****. Contingency table comparing microsatellite status in endometrial cancer.*

|  | **MSI-H** | **MSI-L** | **MSS** |
| --- | --- | --- | --- |
| **Other-Exo** | 1 | 1 | 4 |
| **P286R** | 1 | 5 | 16 |
| **V411L** | 2 | 7 | 4 |

Minor points

9 The authors mention that the P286R amino acid change was predicted to affect the structure of the exonuclease binding pocket. This was actually also demonstrated structurally in the same issue (Parkash et al Nature Comm 2019). In addition to the above, there are a number of curious reference omissions. The 2012 TCGA study that first identified the POLE mutants in colorectal cancer is one. Another is the first reference. There is a vast body of literature on eukaryotic DNA polymerases and their fidelity that might have been used.

We thank the Reviewer for this comment. We have reviewed the references mentioned above and other related references, and have added the necessary information in revised manuscript (**Page 4, and page 14**). As follows:

*“POLE encodes the catalytic subunit of DNA Polymerase Epsilon, which is responsible for DNA fidelity during the process of eukaryotic nuclear genome replication (1). Functional POLE mutations have been identified in less than 1% of all cancer genomes but these genomes are characterized by exceptionally high tumor mutation burden (2). Somatic mutations of POLE exonuclease domain are frequently enriched in brain, uterine and colorectal cancer(3), and patients with POLE dysfunction usually have significantly better prognosis and require less intensive treatment (4).”*

*…*

*“The POLE mutational process shapes the cancer genome into a unique mutational signature with high proportions of C>A mutations at TCT contexts, C>T mutations at TCG contexts and T>G mutations at TTT contexts, which is known as COSMIC signature 10 (4). Several driver mutations have been identified in the POLE exonuclease domain (codons 268–471) (5), the most frequent being P286R and V411L (2). The crystal structure of the yeast orthologue has showed that P301R (P286R in Human) could change the exonuclease domain, with R301 pointing towards the exonuclease site, leading to polymerase hyperactivity and increased capacity to extend mismatches by interfering with DNA binding to the exonuclease site. (6,7). By contrast, residue V411 lies a distance away from the binding site and does not interact with the DNA sequence directly (8). V411L and P286R display different signature fraction with V411L is characterized by relative higher fraction of C>T mutations in endometrial cancer(9).”*

*…*

*“V411L and P286R are the two most frequent POLE mutants and they are located far away from each other in the exonuclease domain, thus conferring different functions (31). Structural and molecular dynamics simulation studies in S. cerevisiae have revealed that P301R (P286R in Human) substitution prevents proper positioning of ssDNA in the exonuclease active site of Pol ε, resulting in promoting the extension of mismatched primer termini (7,8). However, V411 is far away from exonuclease active site may function by affecting the positions of other residues adjacent to the active site (9).”*

10 The pentameric hotspots (e.g. Fig 1D and 3A) would greatly benefit from an inset with the individual sequence contexts clearly labeled.

We thank the Reviewer for this suggestion. The figure would look messy if we label each bar with the context. We indicated the context in a separate table (**Supplementary table 8**), and pointed an arrow and labeled the contexts of interest in the **Fig 1D and Fig 3A.**

• On balance the manuscript would benefit from more careful editing. Several examples through p. 6 include:

Line 50: “cause” should be “caused by”

Line 61: “understand” should be “understanding

Line 89: “due to” is duplicated

Line 136: “Based” should be “Based on”

Thank you for pointing these out, we have now corrected these and further checked out manuscript for such errors.
